# Linking Agent Detection of Invisible Presences to the Self: Relevance for Religious and Spiritual Experiences

**DOI:** 10.3389/fnbeh.2022.952736

**Published:** 2022-06-28

**Authors:** Neza Vehar, Jevita Potheegadoo, Olaf Blanke

**Affiliations:** ^1^Laboratory of Cognitive Neuroscience, Center for Neuroprosthetics and Brain Mind Institute, Ecole Polytechnique Fédérale de Lausanne (EPFL), Geneva, Switzerland; ^2^Department of Clinical Neurosciences, Geneva University Hospital, Geneva, Switzerland

**Keywords:** sensorimotor processing, hallucinations, agent detection, neurology, robotics, anthropology, cognitive science of religion, cultural beliefs

## Introduction

The intriguing experience that somebody is nearby when no one is actually present and cannot be seen or heard has been reported in many different contexts and has been referred to as the sense of presence, feeling of a presence, invisible presences, or presence hallucination (PH) (James, 1902; Critchley, 1979). PHs are often vivid experiences, have a clear location in space—with people frequently turning around to search for the invisible presence—and some even offering it a chair or food (Jaspers, 1913; Nightingale, 1982). PHs are a common theme in fiction, having been alluded to in the literature of divinity, occultism, and parapsychology (Green and McCreery, 1975; Critchley, 1979) and studied in history and anthropology (Solomonova et al., 2011; Wyatt et al., 2016). Following reports of PHs in extreme mountaineering (Smythe, 1935; Messner, 2003), long-distance solo-biking (Davie, 2013), solo-sailing (Suedfeld and Mocellin, 1987) and in shipwreck survivors (Critchley, 1943), PHs have also been investigated in psychology and medicine (Critchley, 1979; Brugger et al., 1996; Arzy et al., 2006). Initially described in psychiatry (Jaspers, 1913; Llorca et al., 2016), PHs have more recently been mostly investigated in neurological patients with epilepsy, stroke, neoplasia, and Parkinson's disease (PD) (Brugger et al., 1996; Fénelon et al., 2011).

However, despite its intriguing experiential characteristics and the broad academic and clinical interest, scientific studies and experimental data on PHs continue to be sparse. This is likely due to difficulties in investigating a spontaneously occurring phenomenon, the absence of experimental procedures able to induce PHs reliably in real time, and to their occurrence in the large majority of cases in situations not prone to empirical investigations (far from laboratories). Here we provide an overview of recent investigations in clinical neuroscience on PH and in neuroscience using methods to induce PH experimentally, linking them to altered self-monitoring and sensorimotor processing. We analyze selected spiritual-religious experiences associated with PH and propose a new extended account of PH, by integrating and extending the altered self-monitoring account with the prominent agent detection theory in spiritual-religious experiences (Guthrie, 1989; Barrett and Lanman, 2008). We conclude by proposing that the mechanism and the controlled induction of invisible presences will likely have an impact in clinical and fundamental neurosciences and may provide a powerful experimental approach in biological anthropology and the cognitive science of religion.

## Neurology and Neuroscience

The feeling of presence has long interested psychologists (James, 1902), psychiatrists (Jaspers, 1913), and neurologists (Critchley, 1979), and has recently also been investigated as a clinical symptom. PH has been reported to co-occur with temporoparietal tumors (Brugger et al., 1996), epilepsy (Critchley, 1979; Brugger et al., 1996), stroke (Blanke et al., 2014), or schizophrenia (Llorca et al., 2016; Stripeikyte et al., 2021). Lately, PH has been classified as a frequent early hallucination in Parkinson's disease (Fénelon et al., 2011; Bernasconi et al., 2021) and Lewy Body dementia (Nagahama et al., 2010). Early evidence about specific brain areas was reported by Arzy et al. (2006), where PH was induced by electrical stimulation in temporoparietal cortex. Interestingly, with repeated stimulations, the PH was perceived with varying attributes (unknown, identified) and with mental states of intentionality and perceived attempts of interference. These data were extended by work using lesion overlap analysis of neurological patients, highlighting the involvement of several cortical regions (Blanke et al., 2014). Due to the high frequency of PH in Parkinson's disease (i.e., Fénelon et al., 2000), a recent study used functional lesion network analysis in patients with Parkinson's disease (Bernasconi et al., 2021) and determined a fronto-temporal PH network, involving ventral premotor cortex, inferior frontal gyrus, and posterior superior temporal sulcus region.

**Figure 1:**
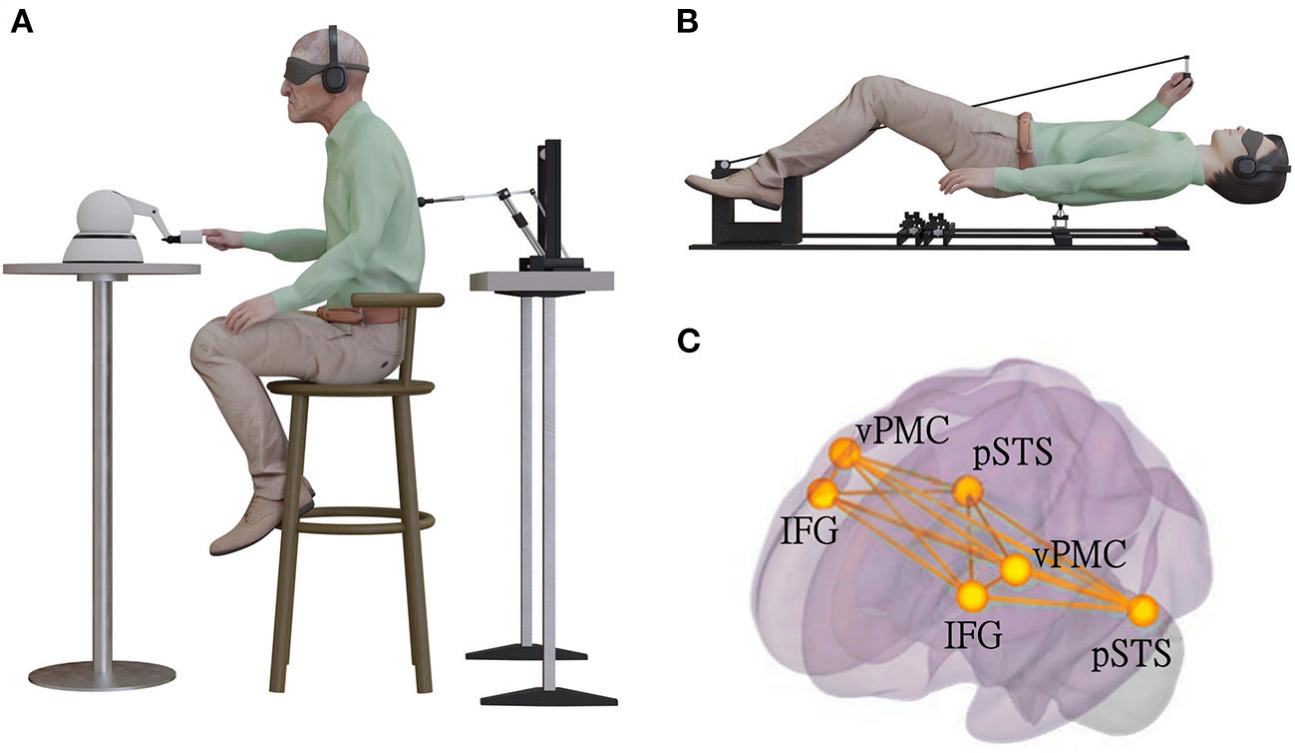
Experimental induction of PH using sensorimotor stimulation (modified after Bernasconi et al., 2021). **(A)** The robotic set-up used for induction of invisible presences in patients with Parkinsons' disease in a sitting position. Patient was moving the front robot in a poking motion, using the right-hand index finger, while receiving a corresponding tactile stimulation on their back. They were in a state of sensory deprivation, wearing headphones playing white noise and with their eyes closed, wearing a blindfold. The stimulation on the back was either synchronous with their movement of the front robot (the back robot had 0 ms of delay) or asynchronous (the back robot randomly delayed from 0 to 500 ms in steps of 100 ms), with the asynchronous stimulation being significantly associated with experiencing robot-induced PH (ri-PH) as a function of sensorimotor delay. **(B)** The robotic set-up was adapted to be MR-compatible and used for an fMRI study in a healthy population of participants. **(C)** The brain activation and connectivity patterns were collected in an fMRI experiment with healthy and neurological non-parkinsonian patients. The schematic bilateral display of the connectivity overlap between the network connectivity in spontaneous PH identified using lesion network and connectivity analysis and ri-PH network from healthy participants. The bilateral regions are ventral premotor cortex (vPMC), inferior frontal gyrus (IFG), and posterior superior temporal sulcus (pSTS).

Based on these data and a prominent model that describes hallucinations as a disturbance or misattribution of self-related predictive sensory signals (Fletcher and Frith, 2009), Blanke et al. (2014) developed a robotic stimulation system that exposed participants to temporally and spatially conflicting sensorimotor signals. Participants were asked to repeatedly move the front robot with their hand (motor, tactile, and proprioceptive signals) and received tactile feedback on their back (back robot), under conditions of sensory deprivation (Figure 1). Being subjected to such conflicting somatosensory-motor stimulations characterized by an additional delay between front and back robot (Bernasconi et al., 2021) elicits PH in healthy individuals (Blanke et al., 2014; Bernasconi et al., 2021; Dhanis et al., 2022). Such robot-induced PH (ri-PH) also allow to study whether certain functions are associated with PH. When ri-PH are elicited while participants carry out a second task, changes in several perceptual and cognitive functions occur, including auditory perception (Orepic et al., 2021), thought insertion (Serino et al., 2021), and cognitive processes (Faivre et al., 2020). Do neurological and ri-PH and the described somatosensory-motor mechanisms relate to presences and invisible agents in anthropology and the science of religion?

## Spiritual-Religious Experiences and PH

The occurrence of invisible or imperceptible supernatural presences is commonly positioned at the core of religious or spiritual belief systems (Luhrmann et al., 2021), with wide cross-cultural variability, ranging from angels, spirits, natural forces to gods. Such presences can be broadly distinguished into intended or voluntary presences (individual actively sought out PH) and unintended, spontaneous presences (individual did not seek to experience PH). Since experiencing supernatural presences is often judged as socially and personally desirable, it is actively sought-after *via* rituals (Otero, 2003; Johnson et al., 2015), ingestion of psychedelic compounds (Sayin, 2016), as well as kindled through training, prayer (Luhrmann and Morgain, 2012; Luhrmann, 2020), or deliberate interaction (Morton, 2020). For example, intended presences have been described by Luhrmann (2012, p. 148) in her anthropological field work with American Evangelicals, actively invoking God's presence (“to feel sensorily aware of God, as if God were a person who was physically present”). Presences with spiritual-religious connotations also happen involuntarily (clinical or non-clinical) and may have transformative effects on a person's life. Non-clinical supernatural presences may “visit-upon” an unsuspecting, healthy person in extreme social or environmental conditions (i.e., Messner, 2003) but may also occur during mundane situations. The latter caught the attention of James (1902), reporting people that “felt a consciousness of a presence in the room (…) not the consciousness of a live person, but of a spiritual presence” (p. 62). James explicitly links PH to the “religious sphere of experience, (where) many persons (…) possess the objects of their belief not in the form of mere conceptions which their intellect accepts as true, but rather in the form of quasi-sensible realities directly apprehended” (p. 64). In the clinical context, PH experiences with spiritual-religious aspects have been noted for a long time. Jaspers (1913) described a patient with schizophrenia who reported PH characterized by “the feeling that the soul of his deceased father is with him,” that the fatherly presence “is behind him” (p. 153), interfering with the patient's life (akin to reports of experiencing presence of ancestral spirits or in the context of grief; Klass and Goss, 1999; Pérez, 2011). Another patient noted at the onset of an epileptic seizure that he felt, “overwhelmingly real,” somebody standing by his side (not seen or heard) and that “God was about to take me home and that I had not to fear anything in the world” (Brugger et al., 1996, p. 116). Despite their different contexts, these reports indicate many PH similarities including spatial aspects (presence behind the person or shoulder, mirroring how angels or other spirits are often represented), psychological attributes of the presence such as strong familiarity, psychological affinity, and identification of presence, and specific intentions (leading a person somewhere; guiding in danger, interfering with a task). PH also mostly appears in low luminosity or contrast conditions, at night and in extreme or monotonous environments, devoid of sensory stimulation—like revelations on mountains (Arzy et al., 2005), Inuit igloo confinement to evoke spirits (Geiger, 2009) or in hermits who retreated to deserts in early Christianity (Suedfeld and Mocellin, 1987).

## Invisible Presences, Hypersensitive Agent Detection and the Cognitive Science of Religion

In brief, Guthrie's anthropomorphism account (Guthrie, 1980, 1989, 2001) linked agent-detection to supernatural beliefs, arguing for a low-level perceptual tendency anthropomorphizing the environment and detecting the presence of humans in environments devoid of others. Generating such false-positive agent perceptions may be adaptive in human evolution, because agents are sources of potential danger or opportunity (Van Leeuwen and van Elk, 2019). Accordingly, it has been argued that PH and supernatural agents (ghosts, gods, spirits) result from the recruitment of hyperactive perceptual mechanisms related to agent-detection. This account on over-detection of *humans* was extended to include *non-human agents* such as animals by Barrett (2000, 2011), and Barrett and Lanman (2008) and the broader detection of *agency* in the environment. As Guthrie's proposal did not account for the intentionality of presences, Barrett and colleagues proposed that agency-detection also involves perception of intentional states (motivations, intentions, desires), beyond mere detection of the presence, based on additional cognitive brain mechanisms (such as mentalizing).

Neuroscience data suggest a different account regarding invisibles presences: altered self-monitoring based on conflicting somatosensory-motor processes involving specific bodily signals. This self-monitoring approach is based on the misperception of oneself as another agent and was tested experimentally with ri-PH: The self is at the origin of invisible presences, being misperceived *as another agent*. We argue that invisible presences result not from visual-auditory mechanisms, as argued previously, but from a different perceptual mechanism: motor signals and their integration with somatosensory signals. These somatosensory-motor signals are specific and involve the global self-representation of a person's body (Blanke and Metzinger, 2009; Park and Blanke, 2019). The self-monitoring approach of PH sides with Guthrie that agent-detection is associated with perception, but primarily results from somatosensory-motor (not visual-auditory) perception, as shown in experiments applying somatosensory-motor stimulation in blindfolded noise-isolated participants (Blanke et al., 2014; Bernasconi et al., 2021; Dhanis et al., 2022; Orepic et al., 2021; Serino et al., 2021). Our proposal refines the anthropomorphic account and supports that the detection of *human agents* (self, global-body representation) and not the broader, less specific detection of *human and non-human agency* (animals, body-part representation) is key in PH. We strongly agree with Barrett that intention recognition, mentalizing, and the notion of minimally counterintuitive states are important to consider in PH. However, the involvement of (too) many different perceptual and cognitive functions conceptually seems to over-complexify matters, hindering empirical verification. Moreover, Barrett's proposed list of additional perceptual and cognitive mechanisms, may not be necessary to perceive invisible agents endowed with intentionality. As reviewed above, perceptual somatosensory-motor mechanisms related to a person's global self-representation are sufficient to perceive an intentional presence and fit well within the category of minimally counterintuitive states (Barrett and Lanman, 2008). Many fascinating questions remain. How does agent detection lead to religious beliefs, how is it shared among kin, and why do humans not simply discard these incorrect perceptions (Boyer, 2001; Van Leeuwen and van Elk, 2019)?

We conclude that the self-monitoring account of invisible presences is relevant not only in neurological and ri-PH, but also anthropology. It is perceptual in nature and links PH to *human agent* detection based on altered somatosensory-motor processing. It differs from previous accounts that have focused on altered perceptual or cognitive mechanisms related to the extrapersonal environment (unrelated to the observer's somatosensory-motor body). Key aspects of supernatural agent detection, as noted by Barrett and Guthrie, are also accounted for by the self-monitoring proposal. We argue that these different accounts are not mutually exclusive: it is rather likely that self-related (egocentric) and environment-related (allocentric) mechanisms are complementary, although we argue that the self-monitoring account is the primary and most basic mechanism of supernatural agent-detection. Finally, the narrative style of both earlier theories has made it difficult to empirically test them, leading to numerous studies with conflicting results (Gervais et al., 2011; van Elk, 2013; van Elk et al., 2016; Maij et al., 2017, 2019). The new method of ri-PH (Blanke et al., 2014; Bernasconi et al., 2021; Serino et al., 2021; Dhanis et al., 2022) provides a promising way to investigate the role of the self in the intriguing human experience of supernatural agents, spirits, and gods. Future studies of invisible presences in different cultural contexts, integrating robot-induced PH with social science approaches, may facilitate the interaction of scholars from neuroscience, anthropology, and the cognitive science of religion.

## Author Contributions

NV and OB contributed to conception and theoretical basis for the article, and wrote sections of the manuscript. NV wrote the first draft of the paper and performed literature review. NV collected cases of PH experiences, under the guidance from OB. JP provided discussion and comments on the manuscript. All authors contributed to manuscript revision, read, and approved the submitted version.

## Funding

The authors are supported by two donors advised by CARIGEST SA (Fondazione Teofilo Rossi di Montelera e di Premuda and a third one wishing to remain anonymous), by National Center of Competence in Research (NCCR Synapsy 51NF40-185897), Parkinson Suisse, and Bertarelli Foundation. This paper is funded by Dr. Rüdiger Seitz, via the Volkswagen Foundation, Siemens Healthineers, and the Betz Foundation. The authors declare that Siemens Healthlineers was not involved in the study design, collection, analysis, interpretation of data, the writing of this article or the decision to submit it for publication.

## Conflict of Interest

OB is an inventor on patent US 10,286,555 B2 (Title: Robot-controlled induction of the feeling of a presence) held by the Swiss Federal Institute (EPFL) that covers the robot-controlled induction of the feeling of a presence (PH). He is an inventor on patent US 10,349,899 B2 (Title: System and method for predicting hallucinations) held by the Swiss Federal Institute (EPFL) that covers a robotic system for the prediction of hallucinations for diagnostic and therapeutic purposes. He is a cofounder and shareholder of Metaphysiks Engineering SA, a company that develops immersive technologies, including applications of the robotic induction of PHs. He is member of the board and shareholder of Mindmaze SA. The remaining authors declare that the research was conducted in the absence of any commercial or financial relationships that could be construed as a potential conflict of interest.

## Publisher's Note

All claims expressed in this article are solely those of the authors and do not necessarily represent those of their affiliated organizations, or those of the publisher, the editors and the reviewers. Any product that may be evaluated in this article, or claim that may be made by its manufacturer, is not guaranteed or endorsed by the publisher.
